# 2-(4-Hy­droxy­phen­yl)acetamide

**DOI:** 10.1107/S2414314625010077

**Published:** 2025-11-14

**Authors:** Allison Kester, Taylor King, Marcus R. Bond

**Affiliations:** a Department of Chemistry and Physics Southeast Missouri State University,Cape Girardeau MO 63701 USA; University of Aberdeen, United Kingdom

**Keywords:** crystal structure, atenolol, acetamino­phen, DFT geometry optimization

## Abstract

In the title mol­ecule, C_8_H_9_NO_2_, which is an isomer of acetamino­phen [*N*-(4-hy­droxy­phen­yl)acetamide], the acetamide group plane subtends a dihedral angle of 89.95 (5)° with respect to the phenyl ring plane with the –NH_2_ group directed outward, in contrast to an *in vacuo* DFT geometry optimization in which the –NH_2_ group is directed inward. In the extended structure, N—H⋯O hydrogen bonds organize mol­ecules into stacks propagating along [100], with additional hydrogen bonding linking neighboring parallel stacks. A survey of known structures indicates that the structures of 2-phenyl­acetamide mol­ecules with any substitution at the 4-position on the phenyl ring can demonstrate different orientations for the acetamide group ranging from the –NH_2_ group directed almost completely outward to the –NH_2_ group directed almost completely inward.

## Abstract 2

In the title mol­ecule, an isomer of acetamino­phen, the acetamide plane is perpendicular to the phenyl ring plane with the –NH_2_ group directed outward, in contrast to an *in vacuo* DFT geometry optimization in which the –NH2 group is directed inward.

## Graphical abstract

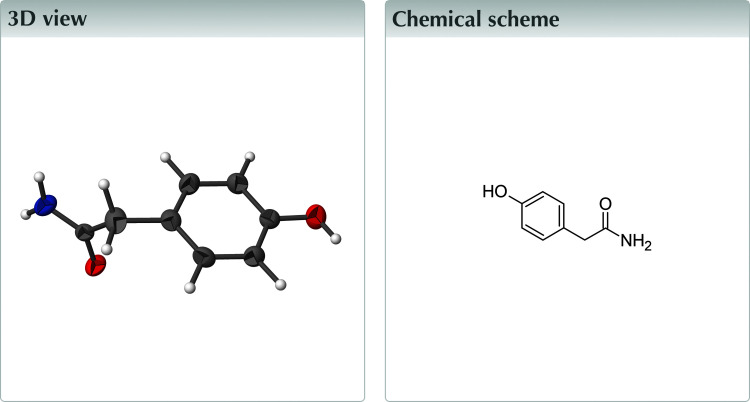

## Structure description

**Figure 1:**
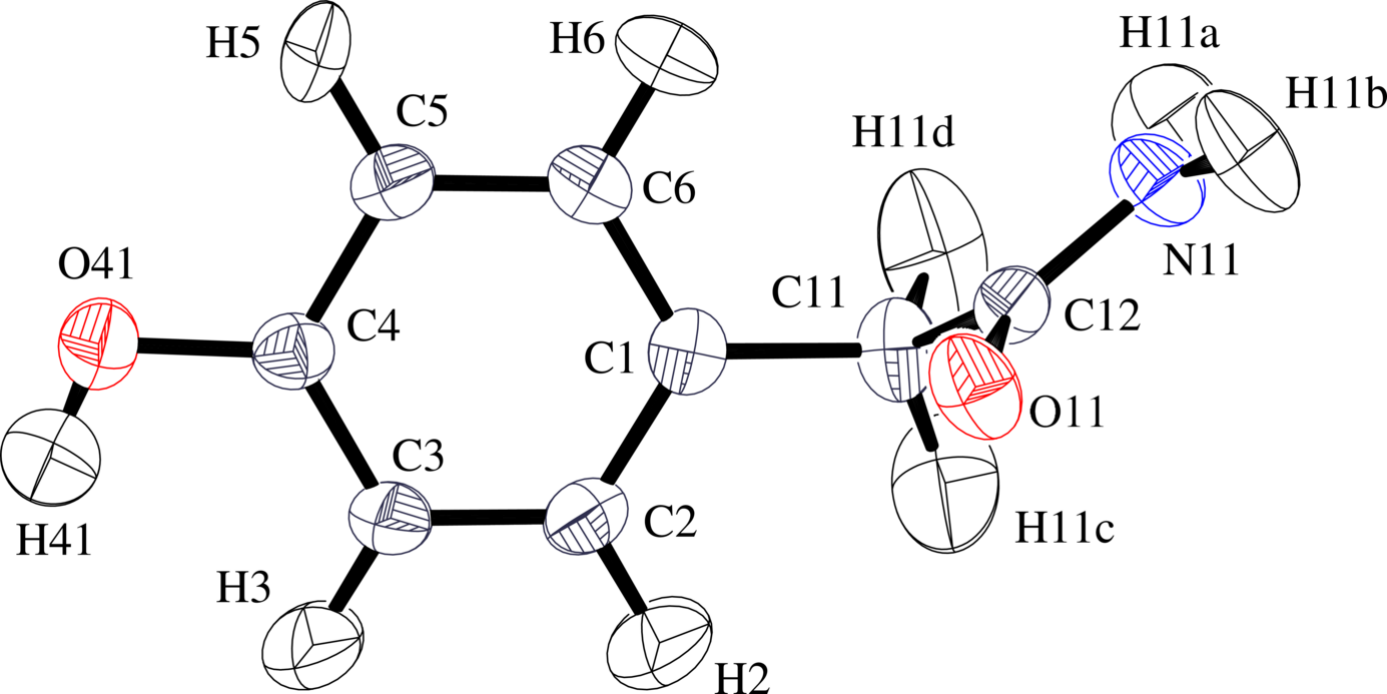
Displacement ellipsoid plot of (**I**) at the 50% level with labels for all atoms.

The title mol­ecule, C_8_H_9_NO_2_ (**I**), is an isomer of *N*-(4-hy­droxy­phen­yl)acetamide [Cambridge Structural Database (CSD) refcodes: HXACAN01–67], also known as acetamino­phen or paracetamol in different countries. The mean C_a_—N (a = amide) bond length in structures of acetamino­phen calculated from values in the CSD [1.346 (25) Å] is 0.026 Å longer than in (**I**) (Fig. 1 ▸). The short C—N bond length in (**I**) is consistent with the well known ‘amide resonance’ effect (Kemnitz & Loewen, 2007 ▸). Bond lengths and angles within the amide group of (**I**) agree with mean values found for 2-substituted acetamide groups in the CSD [C—N/=O: 1.32 (5)/1.23 (5) Å; N—C=O/C—C=O/C—C—N: 122 (5)/121 (5)/116 (5)°; 1620 hits, CSD Version 5.00, August 2025 updates, Groom *et al.*, 2016 ▸). The –NH_2_ group in (**I**) is almost planar with slight pyramidalization [the N atom lies 0.017 Å above the C12/N11/(H1*a*, H1*b*) mean plane] so that *sp*^2^ hybridization can be assigned to N11. The C_ar_—OH (ar = aromatic) bond length [1.381 (3) Å] found in the structure of the parent acid, 4-hy­droxy­phenyl­acetic acid (QAPBAL; Gracin & Fischer, 2005 ▸) is some 0.019 Å longer than in (**I**). The C_ar_—O—H angle agrees within 1 s.u. of 109.5° so that *sp*^3^ hybridization can be assume for O41.

**Figure 2:**
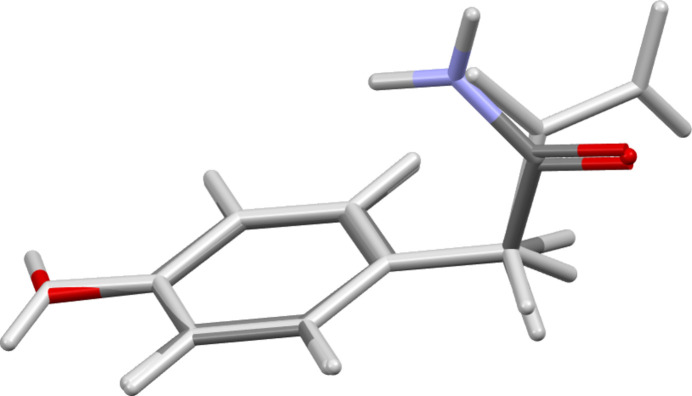
Capped stick plots of the DFT-optimized geometry (color scheme: C, gray; H, white; N, blue; O, red) superimposed on the experimental geometry (light gray) of (**I**).

**Figure 3:**
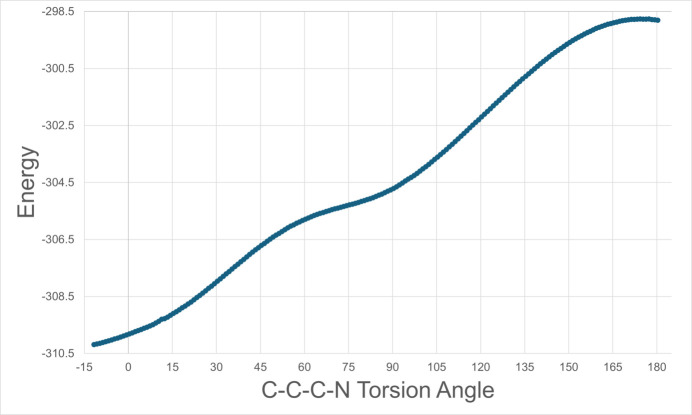
Plot of a semiempirical, partially relaxed scan in 1° increments of the C1—C11—C12—N11 torsion angle in (**I**) starting at the DFT-optimized value to a value of 180.3°. The acetamide group is constrained to be approximately planar and to be approximately perpendicular to the phenyl ring during the scan. Energy (kJ mol^−1^) is plotted on the vertical axis with the torsion angle (°) plotted on the horizontal axis.

The core atoms of (**I**) (C1–C6/C11/O41) are effectively planar [root-mean-square deviation (RMSD) = 0.003 Å] as are those of the acetamide group (C11, C12, O11, and N11; RMSD = 0.005 Å) with mean plane normals perpendicular [89.98 (5)°]. The –NH_2_ group is directed outward from the phenyl ring and the carboxyl O-atom directed inward but not fully, as shown by the C1—C11—C12—N11 torsion angle of −135.91 (12)°. In contrast, since the N atom is bound to the phenyl ring in acetamino­phen, the acetamide plane and the core atom plane are more closely aligned, *e.g.* with angles of 20.56 (5)° (monoclinic form I, HXACAN64) and 16.97 (5)° (ortho­rhom­bic form II, HXACAN65) between mean plane normals in recent structure determinations (Weatherston, 2024 ▸). In QAPBAL, the mean plane of the acid group is almost perpendicular to the phenyl ring mean plane [93.22 (14)°]. Here the carboxyl O-atom is directed inward, but with a larger torsion angle magnitude [159.8 (3)°]. A DFT geometry optimization [B3LYP, 6311+G(d,p); GAMESS (Schmidt *et al.*, 1993 ▸)] of (**I**) *in vacuo* results in the acetamide plane almost perpendicular (90.33°) to the plane of the core atoms, but with the carboxyl O atom directed outward, the –NH_2_ group directed inward, and a torsion angle of −11.68° (Fig. 2 ▸). A semi-empirical, partially relaxed scan of this torsion angle (MOPAC2016, Version 19.255*L*, PM7 Hamiltonian; Stewart, 2016 ▸) shows a steady rise in energy from its optimized value to a maximum value as the torsion angle approaches 180° (Fig. 3 ▸). A MOL file of the optimized geometry has been placed in the supporting information.

A search of the CSD for 2-phenyl­acetamide mol­ecules with any substitution at the 4-position on the phenyl ring yielded 21 hits that are dominated by pharmaceutically related compounds or natural products. Ten of these are structures of atenolol (CEZVIN and CIDHAZ; de Castro *et al.*, 2007 ▸), a β blocker medication for treatment of high blood pressure (Heel *et al.*, 1979 ▸), or its salts or cocrystal: [succinate (DETHIU; Cai *et al.*, 2006 ▸), nicotinate and isonicotinate (GUJBOG and GUJCAT; Botes *et al.*, 2024 ▸), fumarate and adipate (IGUWUG and UHOGUX; Shajan *et al.*, 2024 ▸), 4-amino­benzoate (JIRWIR; Lou *et al.*, 2007 ▸), chloride (WEWLOC; Rama Kumar *et al.*, 2018 ▸), and bi­naphthyl­phosphate (QAJYIL; Wang & Chen, 2011 ▸)]. The atenolol mol­ecule possesses a substituted prop­oxy group at the 4-position and, as a result, (**I**) is a common reagent in its synthetic preparation (Procopio *et al.*, 2024 ▸).

**Figure 4:**
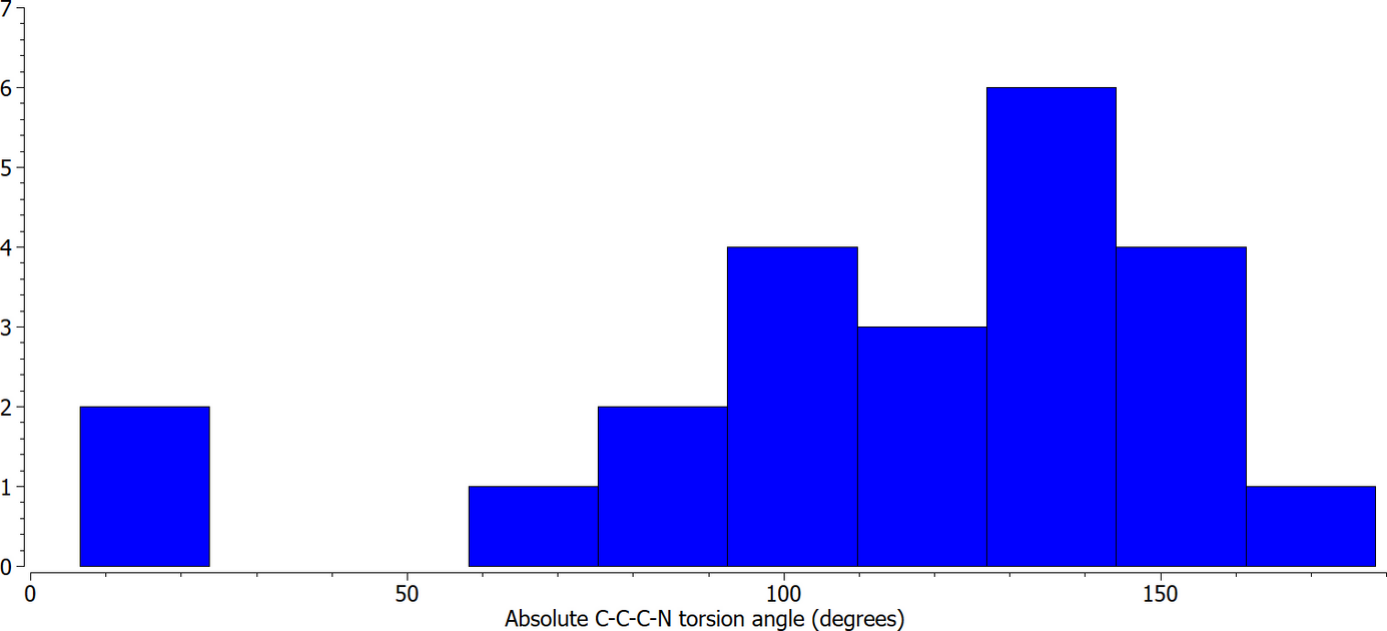
Histogram of the C—C—C—N torsion angle magnitude frequency for 2-phenyl­acetamide mol­ecules with any substitution at the 4-position on the phenyl ring.

The crystal structures of atenolol show an orientation for the acetamide group similar to that in (**I**), *i.e.*, approximately perpendicular inter­planar angles [86.01 (9)° for CEZVIN, 90.43 (11) and 86.74 (11)° for CIDHAZ] and similar torsion angle magnitudes [141.24 (19)° for CEZVIN, 135.0 (3) and 142.1 (2)° for CIDHAZ]. The acetamide groups in the salts and cocrystal show a range of orientations. For GUJBOG, JIRWIR, UHOGUX, and WEWLOC, the –NH_2_ group is directed more outward, for DETHIEU, GUJCAT, and IGUWUG neither the –NH_2_ nor –C=O groups are directed outward significantly, while for QAJYIL the –NH_2_ group is directed more inward. In the structures of two other compounds, the natural product millingtojanine A (BAKWUJ; Jumai *et al.*, 2021 ▸) and 2-carboxamido­methyl-4,5-dimeth­oxy-phenyl-*N*,*N*-di­ethyl­sulfonamide (CXMESX; Hamodrakas *et al.*, 1977 ▸), the amide group is directed almost completely inward and similar to the orientation found in the DFT geometry optimization. At the opposite extreme is the structure of 2-(4-chloro­phen­yl)acetamide (OCETAT; Ma *et al.*, 2011 ▸) in which the –NH_2_ group is directed almost completely outward [torsion angle = 178.6 (2)°]. A histogram of torsion angle magnitudes for these compounds (Fig. 4 ▸) shows the full range of –NH_2_ group orientations with a mean value of 115° (standard deviation = 42°) and a median of 121.7°. The orientation of the acetamide group appears to depend on competition between minimizing the mol­ecular energy and optimizing the inter­molecular hydrogen-bonding inter­actions, *e.g.*, an outward-directed –NH_2_ group may be more available as a hydrogen-bond donor if a suitable acceptor atom is present.

**Figure 5:**
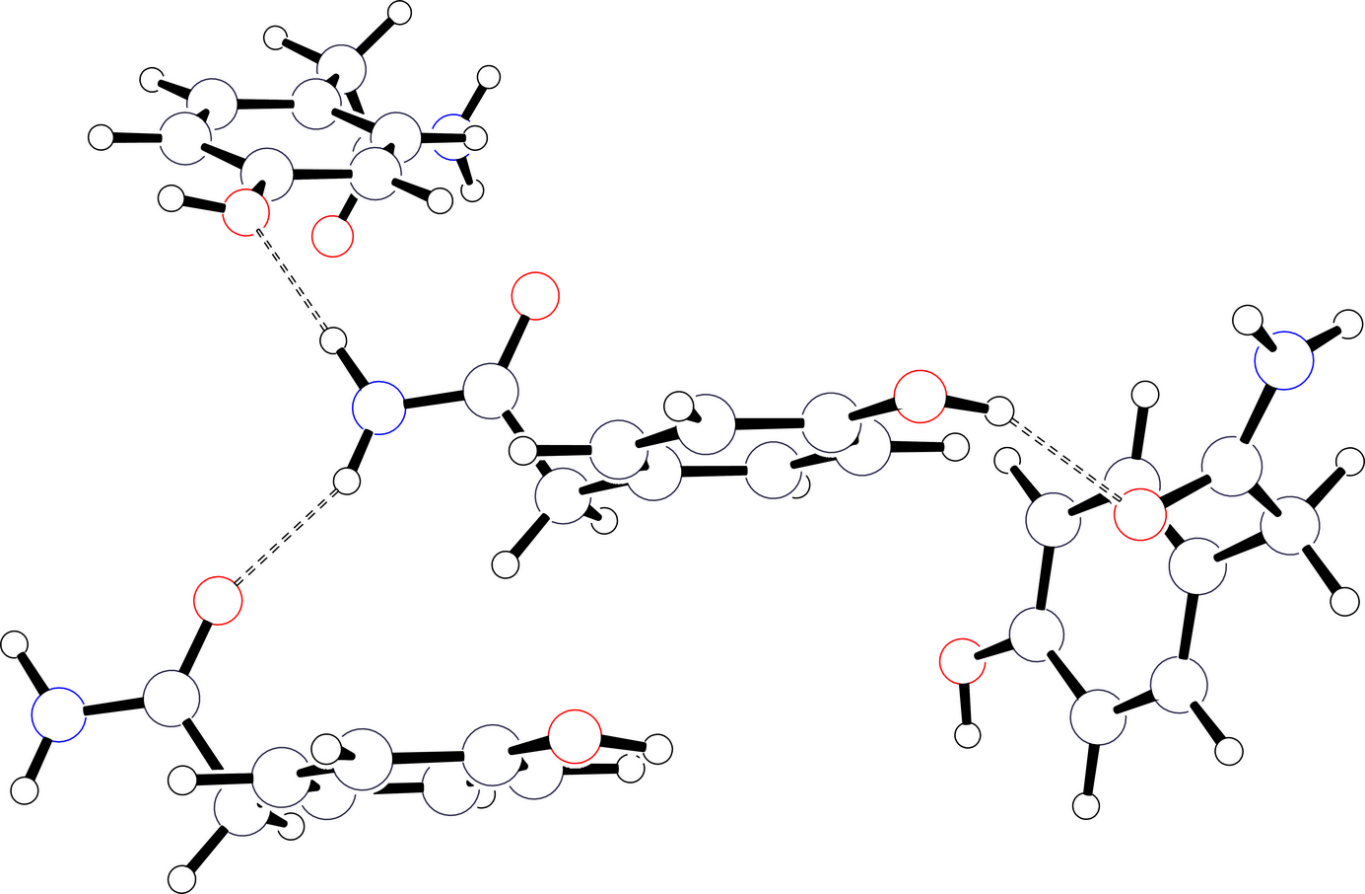
Donor hydrogen-bond inter­actions by a given mol­ecule in (**I**) to three neighboring mol­ecules. Atoms are drawn as circles of arbitrary radii and hydrogen bonds are indicated by dashed lines.

**Figure 6:**
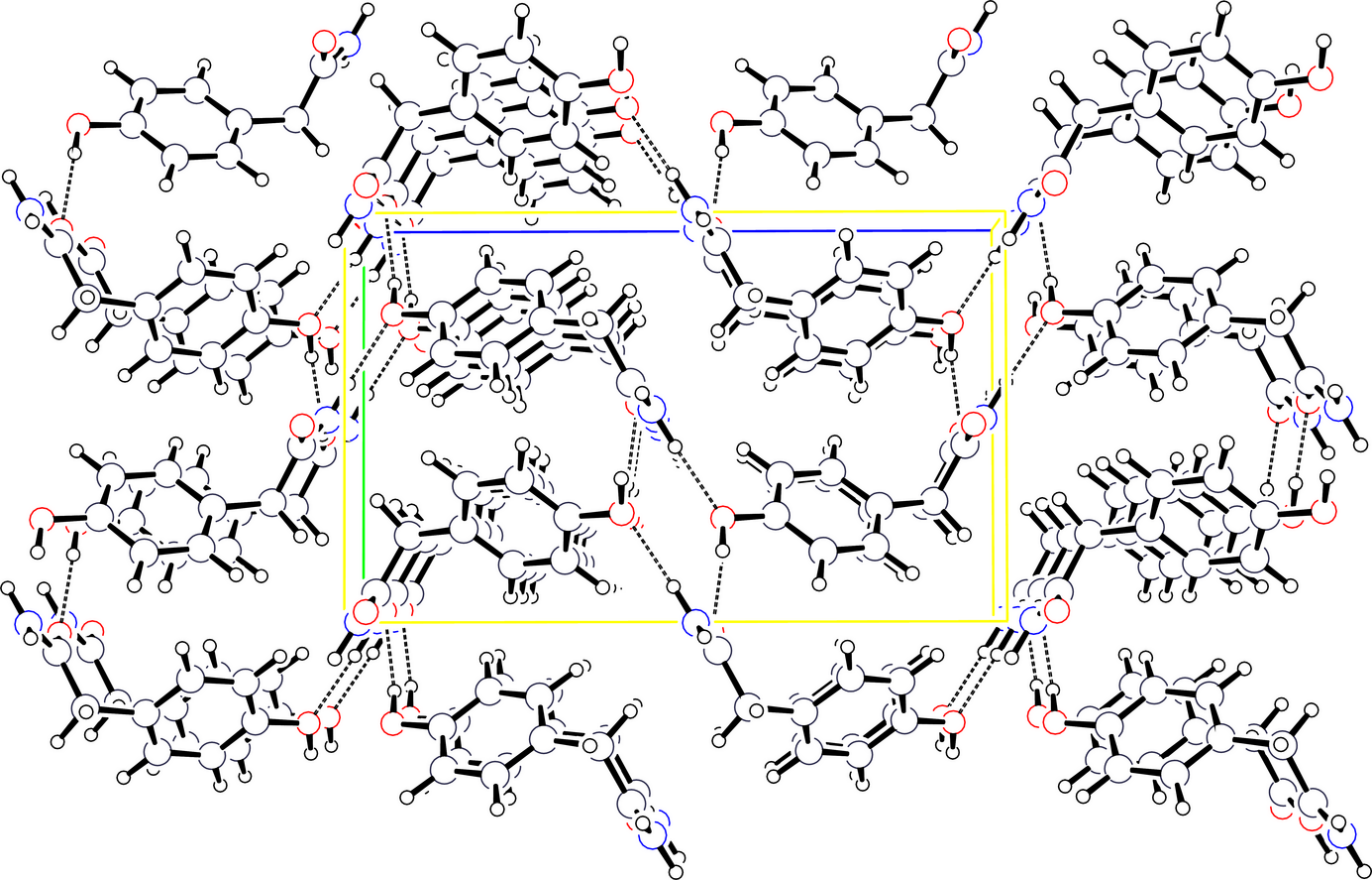
Unit-cell packing diagram for (**I**) viewed down *a* with *b* vertical and *c* horizontal. Atoms are drawn as circles of arbitrary radii and hydrogen bonds are indicated by dashed lines.

The –NH_2_ group in (**I**) is a hydrogen-bond donor to carboxyl and to hydroxyl O atoms while the hydroxyl group is a hydrogen-bond donor to a carboxyl O atom, each to different mol­ecules (Fig. 5 ▸). The N—H⋯O=C inter­action links mol­ecules into stacks along *a* with the other inter­actions linking neighboring parallel stacks (Fig. 6 ▸). By comparison, OCETAT is found in the same space group as (**I**) and with a slightly larger mol­ecular volume [197.9 (2) Å^3^*versus* 186.12 (2) Å^3^ in (**I**)], but with the chloro substituent not involved in N—H hydrogen bonding. In this case, the extended structure consists of herringbone bilayers with the acetamide groups linked by N—H⋯O hydrogen bonding on the outside of the bilayer while the 4-chloro substituents abut each other in the middle. Hydrogen-bond geometrical data for (**I**) are presented in Table 1 ▸.

## Synthesis and crystallization

2-(4-Hy­droxy­phen­yl)acetamide (Aldrich, 99%) was dissolved in methanol and diffraction-quality crystals grown by slow evaporation at room temperature.

## Refinement

Crystal data, data collection, and structure refinement details are listed in Table 2 ▸. Structure solution and initial refinement using an independent atom model occurred within the Bruker *APEX3* software package (Version 2019/11–0; Bruker 2019 ▸) followed by Hirshfeld atom refinement within the *OLEX2*–1.5 system using *NoSpherA2* (Kleemiss *et al.*, 2021 ▸; Midgley *et al.*, 2021 ▸). Non-spherical atomic form factors were derived from electron density determined by DFT calculations using *ORCA 5.0* (B3LYP functional, def2-SVP basis set; Neese, 2022 ▸). All atoms were refined anisotropically. Two low angle reflections with *F*_o_*<< F*_c_ were presumed to be blocked by the beam catcher and omitted from the refinement. A secondary extinction correction coefficient was refined to a value of 0.017 (2).

## Supplementary Material

Crystal structure: contains datablock(s) I. DOI: 10.1107/S2414314625010077/hb4542sup1.cif

Structure factors: contains datablock(s) I. DOI: 10.1107/S2414314625010077/hb4542Isup2.hkl

MOL file for DFT geometry optimized molecule. DOI: 10.1107/S2414314625010077/hb4542sup3.mol

Supporting information file. DOI: 10.1107/S2414314625010077/hb4542Isup4.cml

CCDC reference: 2502168

Additional supporting information:  crystallographic information; 3D view; checkCIF report

## Figures and Tables

**Table 1:** Hydrogen-bond geometry (Å, °)

*D*—H⋯*A*	*D*—H	H⋯*A*	*D*⋯*A*	*D*—H⋯*A*
O41—H41⋯O11^i^	0.914 (18)	1.92 (2)	2.7703 (13)	154.1 (16)
N11—H11*a*⋯O11^ii^	0.947 (16)	2.041 (16)	2.9365 (16)	157.1 (13)
N11—H11*b*⋯O41^iii^	1.000 (15)	1.965 (16)	2.9646 (19)	178.0 (13)

Symmetry codes: (i) 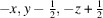; (ii) 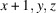; (iii) 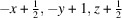.

**Table 2:** Experimental details

Crystal data	
Chemical formula	C_8_H_9_NO_2_
*M*_r_	151.17
Crystal system, space group	Orthorhombic, *P*2_1_2_1_2_1_
Temperature (K)	295
*a*, *b*, *c* (Å)	5.0935 (2), 9.5089 (4), 15.3708 (7)
*V* (Å^3^)	744.46 (6)
*Z*	4
Radiation type	Mo *K*α
μ (mm^−1^)	0.10
Crystal size (mm)	0.49 × 0.21 × 0.17

Data collection	
Diffractometer	Bruker D8 Quest Eco CCD
Absorption correction	Multi-scan (*SADABS*; Krause *et al.*, 2015 ▸)
*T*_min_, *T*_max_	0.689, 0.746
No. of measured, independent and observed [*I* ≥ 2u(*I*)] reflections	21517, 1908, 1483
*R*_int_	0.057
(sin θ/λ)_max_ (Å^−1^)	0.675

Refinement	
*R*[*F*^2^ > 2σ(*F*^2^)], *wR*(*F*^2^), *S*	0.038, 0.048, 1.13
No. of reflections	1908
No. of parameters	182
H-atom treatment	All H-atom parameters refined
Δρ_max_, Δρ_min_ (e Å^−3^)	0.20, −0.21
Absolute structure	Hooft *et al.* (2010 ▸)
Absolute structure parameter	−0.1 (5)

Computer programs: *SAINT* (Bruker, 2019 ▸), *SHELXT2018/2* (Sheldrick, 2015*a* ▸), *OLEX2.refine* (Bourhis *et al.*, 2015 ▸), *SHELXL2018/3* (Sheldrick, 2015*b* ▸), *ORTEP-3 for Windows* (Farrugia, 2012 ▸), *Mercury* (Macrae *et al.*, 2020 ▸) and *publCIF* (Westrip, 2010 ▸).

## full crystallographic data

### 2-(4-Hydroxyphenyl)acetamide Crystal data

**Table:**

C_8_H_9_NO_2_	*D*_x_ = 1.349 Mg m^−^^3^
*M**_r_* = 151.17	Mo *K*α radiation, λ = 0.71073 Å
Orthorhombic, *P*2_1_2_1_2_1_	Cell parameters from 6683 reflections
*a* = 5.0935 (2) Å	θ = 3.4–26.3°
*b* = 9.5089 (4) Å	µ = 0.10 mm^−^^1^
*c* = 15.3708 (7) Å	*T* = 295 K
*V* = 744.46 (6) Å^3^	Rod, colourless
*Z* = 4	0.49 × 0.21 × 0.17 mm
*F*(000) = 320.229	

### 2-(4-Hydroxyphenyl)acetamide Data collection

**Table:**

Bruker D8 Quest Eco CCD diffractometer	1483 reflections with *I*≥ 2u(*I*)
φ and ω scans	*R*_int_ = 0.057
Absorption correction: multi-scan (SADABS; Krause *et al.*, 2015)	θ_max_ = 28.7°, θ_min_ = 3.4°
*T*_min_ = 0.689, *T*_max_ = 0.746	*h* = −6→6
21517 measured reflections	*k* = −12→12
1908 independent reflections	*l* = −20→20

### 2-(4-Hydroxyphenyl)acetamide Refinement

**Table:**

Refinement on *F*^2^	All H-atom parameters refined
Least-squares matrix: full	*w* = 1/[σ^2^(*F*_o_^2^) + (0.0163*P*)^2^ + 0.0113*P*] where *P* = (*F*_o_^2^ + 2*F*_c_^2^)/3
*R*[*F*^2^ > 2σ(*F*^2^)] = 0.038	(Δ/σ)_max_ = 0.001
*wR*(*F*^2^) = 0.048	Δρ_max_ = 0.20 e Å^−^^3^
*S* = 1.13	Δρ_min_ = −0.20 e Å^−^^3^
1908 reflections	Extinction correction: Zachariasen, Fc^*^=kFc[1+0.001xFc^2^λ^3^/sin(2θ)]^-1/4^
182 parameters	Extinction coefficient: 0.017 (2)
0 restraints	Absolute structure: Hooft *et al.* (2010)
0 constraints	Absolute structure parameter: −0.1 (5)
Primary atom site location: dual	

### 2-(4-Hydroxyphenyl)acetamide Fractional atomic coordinates and isotropic or equivalent isotropic displacement parameters (Å^2^)

**Table:**

	*x*	*y*	*z*	*U*_iso_*/*U*_eq_	
O11	0.22820 (14)	0.47323 (8)	0.44521 (5)	0.0404 (2)	
O41	−0.0450 (2)	0.24609 (10)	0.07225 (7)	0.0437 (3)	
H41	−0.152 (3)	0.169 (2)	0.0717 (11)	0.069 (6)	
N11	0.6580 (3)	0.49778 (15)	0.46884 (8)	0.0407 (3)	
H11a	0.829 (3)	0.4650 (16)	0.4559 (9)	0.057 (4)	
H11b	0.620 (3)	0.5855 (16)	0.5025 (10)	0.054 (5)	
C1	0.3607 (2)	0.27802 (11)	0.30625 (6)	0.0329 (3)	
C2	0.1609 (2)	0.18008 (13)	0.29703 (8)	0.0360 (3)	
H2	0.111 (3)	0.1155 (13)	0.3516 (8)	0.065 (4)	
C3	0.0224 (3)	0.16737 (13)	0.21944 (8)	0.0374 (3)	
H3	−0.129 (3)	0.0902 (14)	0.2123 (7)	0.068 (4)	
C4	0.0839 (2)	0.25385 (11)	0.14971 (7)	0.0326 (3)	
C5	0.2843 (3)	0.35193 (14)	0.15763 (9)	0.0391 (3)	
H5	0.332 (3)	0.4167 (14)	0.1032 (7)	0.064 (4)	
C6	0.4203 (3)	0.36305 (14)	0.23543 (8)	0.0396 (3)	
H6	0.573 (3)	0.4385 (14)	0.2415 (9)	0.076 (5)	
C11	0.5106 (3)	0.29262 (15)	0.39025 (9)	0.0405 (3)	
H11c	0.459 (3)	0.2104 (13)	0.4351 (9)	0.085 (6)	
H11d	0.716 (3)	0.2869 (16)	0.3787 (9)	0.084 (5)	
C12	0.4544 (2)	0.42959 (11)	0.43655 (7)	0.0283 (3)	

### 2-(4-Hydroxyphenyl)acetamide Atomic displacement parameters (Å^2^)

**Table:**

	*U*^11^	*U*^22^	*U*^33^	*U*^12^	*U*^13^	*U*^23^
O11	0.0233 (4)	0.0482 (5)	0.0498 (5)	0.0059 (4)	−0.0030 (4)	−0.0130 (4)
O41	0.0497 (6)	0.0443 (6)	0.0370 (5)	−0.0048 (5)	−0.0088 (5)	0.0048 (5)
H41	0.054 (12)	0.097 (15)	0.055 (11)	−0.021 (11)	−0.004 (10)	0.011 (12)
N11	0.0260 (6)	0.0446 (8)	0.0517 (7)	0.0003 (6)	−0.0017 (6)	−0.0046 (6)
H11a	0.040 (9)	0.071 (10)	0.062 (10)	0.011 (9)	−0.003 (9)	0.008 (9)
H11b	0.043 (10)	0.042 (9)	0.077 (12)	−0.001 (8)	−0.013 (8)	−0.026 (8)
C1	0.0345 (6)	0.0314 (6)	0.0328 (6)	0.0065 (6)	0.0001 (5)	−0.0032 (5)
C2	0.0413 (7)	0.0353 (7)	0.0313 (7)	−0.0011 (6)	0.0057 (6)	0.0027 (6)
H2	0.088 (11)	0.057 (9)	0.050 (8)	−0.018 (9)	−0.001 (8)	0.020 (7)
C3	0.0400 (7)	0.0362 (8)	0.0360 (7)	−0.0103 (6)	0.0014 (6)	0.0010 (6)
H3	0.094 (11)	0.062 (10)	0.047 (8)	−0.035 (10)	−0.002 (8)	0.012 (7)
C4	0.0343 (7)	0.0295 (6)	0.0339 (6)	−0.0007 (6)	0.0023 (5)	−0.0003 (5)
C5	0.0460 (8)	0.0381 (7)	0.0332 (7)	−0.0083 (6)	0.0013 (6)	0.0050 (6)
H5	0.082 (10)	0.075 (10)	0.034 (7)	−0.025 (9)	−0.027 (8)	0.020 (7)
C6	0.0402 (8)	0.0379 (7)	0.0407 (8)	−0.0100 (6)	−0.0024 (6)	0.0005 (5)
H6	0.097 (12)	0.066 (9)	0.065 (9)	−0.057 (10)	−0.013 (9)	0.008 (8)
C11	0.0396 (9)	0.0395 (8)	0.0424 (8)	0.0136 (7)	−0.0090 (7)	−0.0042 (6)
H11c	0.137 (16)	0.037 (8)	0.080 (11)	−0.002 (9)	−0.042 (11)	0.015 (9)
H11d	0.071 (11)	0.091 (12)	0.090 (12)	0.038 (11)	−0.006 (9)	−0.059 (9)
C12	0.0237 (6)	0.0342 (6)	0.0270 (6)	0.0037 (5)	0.0007 (5)	0.0022 (5)

### 2-(4-Hydroxyphenyl)acetamide Geometric parameters (Å, º)

**Table:**

O11—C12	1.2319 (12)	C2—C3	1.3908 (16)
O41—H41	0.914 (18)	C3—H3	1.072 (13)
O41—C4	1.3618 (15)	C3—C4	1.3868 (15)
N11—H11a	0.947 (16)	C4—C5	1.3878 (16)
N11—H11b	1.000 (15)	C5—H5	1.067 (11)
N11—C12	1.3197 (16)	C5—C6	1.3862 (18)
C1—C2	1.3869 (16)	C6—H6	1.062 (13)
C1—C6	1.3895 (16)	C11—H11c	1.075 (14)
C1—C11	1.5063 (17)	C11—H11d	1.065 (15)
C2—H2	1.069 (11)	C11—C12	1.5115 (17)
			
C4—O41—H41	109.9 (11)	H5—C5—C4	119.1 (7)
H11b—N11—H11a	124.2 (14)	C6—C5—C4	119.63 (12)
C12—N11—H11a	118.8 (9)	C6—C5—H5	121.3 (7)
C12—N11—H11b	116.8 (9)	C5—C6—C1	121.48 (12)
C6—C1—C2	118.11 (11)	H6—C6—C1	118.9 (8)
C11—C1—C2	121.42 (11)	H6—C6—C5	119.6 (8)
C11—C1—C6	120.47 (12)	H11c—C11—C1	110.9 (7)
H2—C2—C1	118.6 (7)	H11d—C11—C1	110.6 (8)
C3—C2—C1	121.20 (12)	H11d—C11—H11c	108.2 (11)
C3—C2—H2	120.2 (7)	C12—C11—C1	112.78 (10)
H3—C3—C2	120.9 (6)	C12—C11—H11c	106.1 (7)
C4—C3—C2	119.78 (12)	C12—C11—H11d	108.0 (8)
C4—C3—H3	119.3 (6)	N11—C12—O11	121.91 (12)
C3—C4—O41	122.32 (11)	C11—C12—O11	121.20 (11)
C5—C4—O41	117.89 (11)	C11—C12—N11	116.86 (12)
C5—C4—C3	119.79 (11)		
			
O11—C12—C11—C1	45.69 (11)	C2—C1—C11—C12	−111.31 (12)
O41—C4—C3—C2	−179.97 (11)	C2—C3—C4—C5	0.39 (14)
O41—C4—C5—C6	−179.99 (11)	C3—C2—C1—C6	−0.34 (13)
N11—C12—C11—C1	−135.91 (12)	C3—C2—C1—C11	179.75 (11)
C1—C2—C3—C4	−0.05 (13)	C3—C4—C5—C6	−0.33 (13)
C1—C6—C5—C4	−0.07 (14)	C5—C6—C1—C11	−179.69 (11)
C2—C1—C6—C5	0.40 (13)	C6—C1—C11—C12	68.78 (12)

### 2-(4-Hydroxyphenyl)acetamide Hydrogen-bond geometry (Å, º)

**Table:**

*D*—H···*A*	*D*—H	H···*A*	*D*···*A*	*D*—H···*A*
O41—H41···O11^i^	0.914 (18)	1.92 (2)	2.7703 (13)	154.1 (16)
N11—H11*a*···O11^ii^	0.947 (16)	2.041 (16)	2.9365 (16)	157.1 (13)
N11—H11*b*···O41^iii^	1.000 (15)	1.965 (16)	2.9646 (19)	178.0 (13)

Symmetry codes: (i) −*x*, *y*−1/2, −*z*+1/2; (ii) *x*+1, *y*, *z*; (iii) −*x*+1/2, −*y*+1, *z*+1/2.
